# Retraction: Thin layer drying kinetics and quality dynamics of persimmon (*Diospyros kaki*) treated with preservatives and solar dried under different temperatures

**DOI:** 10.1371/journal.pone.0275326

**Published:** 2022-09-22

**Authors:** The PLOS ONE Editors

This article [1] was identified as one of a series of submissions for which we have concerns about authorship, competing interests, and peer review.

In addition, Fig 1 and the information in the “Flat plate solar drier” Methods subsection were previously published by a different group in [2]. The 2016 article was not cited or discussed as a source of this material in the *PLOS ONE* article.

In light of these issues, the *PLOS ONE* Editors retract this article. We regret that the issues were not addressed prior to the article’s publication.

AK and RAY did not agree with retraction. The other authors did not reply or could not be reached.

As discussed above, Fig 1 of [1] reports material from [2], published 2016 [Sains Malaysiana], which is not offered under a CC-BY license. Fig 1 is therefore excluded from this article’s [1] license. At the time of retraction, the article [1] was republished to note this exclusion in the Fig 1 legend and the article’s copyright statement.
